# Cognitive adverse effects of epilepsy and its predictors attending outpatient department of South Gondar zone hospitals, Amhara Region, Ethiopia 2020 /2021

**DOI:** 10.1371/journal.pone.0278908

**Published:** 2022-12-09

**Authors:** Sintayehu Asnakew, Getasew Legas, Amsalu Belete, Fitalew Tadele Admasu, Getachew Yideg Yitbarek, Tigabu Munye Aytenew, Biruk Demise, Eshetie Molla Alemu, Muluken Adela Alemu, Wubet Alebachew Bayih, Dejen Getaneh Feleke, Ermias Sisay Chanie, Binyam Munye Birhane, Demewoz Kefale

**Affiliations:** 1 Department of Psychiatry, School of Medicine, College of Health Science, Debre Tabor University, Debre Tabor, Ethiopia; 2 Department of Biomedical Sciences, College of Health Science, Debre Tabor University, Debre Tabor, Ethiopia; 3 Department of Nursing, College of Health Science Debre Tabor University, Debre Tabor, Ethiopia; 4 Departments of Social and Population Health, College of Health Science, Debre Tabor University, Debre Tabor, Ethiopia; 5 Departemnt of Pharmacy, College of Health Science, Debre Tabor University, Debre Tabor, Ethiopia; 6 Department of Pediatrics and Child Health and Neonatal Nursing, College of Health Science, Debre Tabor University, Debre Tabor, Ethiopia; UCSI University, MALAYSIA

## Abstract

**Background:**

Epilepsy is the most common neurologic disorder which is further complicated by neurobehavioral co-morbidities, cognitive impairment, psychiatric disorders, and social problems. However, assessments of cognitive status of epileptic patients are far too low during clinical visits. This calls for early neuropsychological assessment soon after the diagnosis of epilepsy for a better treatment plan and outcome for epileptic patients.

**Objective:**

This study aimed to assess the cognitive adverse effects of epilepsy and its predictors attending outpatient departments of South Gondar Zone hospitals Amhara region Ethiopia 2020/2021.

**Methods:**

A multi-center institutional-based cross-sectional study was conducted. A total of 509 respondents were included with a response rate of 93.9%. Previously adapted pretested structured questionnaire was used containing, socio-demographic, clinical, and seizure related factors. Mini-Mental State Examination (MMSE) was used to measure cognitive impairment. A systematic random sampling technique was applied. Data were entered into Epi data version 4.4.2 then exported to SPSS version 24 for analysis. Descriptive statistics, bivariable and multivariable binary logistic regressions with odds ratios and 95% confidence interval were employed. The level of significance of association was determined at a p-value < 0.05.

**Results:**

Prevalence of cognitive impairment in this study was 69.2% (95%CI; 65.4, 73.1). Rural residents (AOR = 4.16,95%CI, 1.99,8.67), respondents who couldn’t read and write (AOR = 2.62, 95%CI; 1.24, 5.5,) longer duration of seizure disorder (AOR = 4.59,95%CI; 2.01,10.52), taking combined Phenobarbital and Phenytoin (AOR = 4.69,95%CI; 1.88,11.69), having history of head injury (AOR = 3.29,95%CI;1.30,8.32), having depression (AOR = 4.76,95%CI;2.83,7.98), and anxiety (AOR = 3.11,95%CI; 1.58,6.12) were significantly associated with cognitive impairment.

**Conclusions:**

Prevalence of cognitive impairment in this study was high. Regular neuropsychiatric assessment of patients with epilepsy should be encouraged especially for those participants with longer durations of illness, who are rural residents, who take combined Phenobarbital and Phenytoin, participants who had a history of head injury, depression, and anxiety.

## Introduction

Epilepsy is a neurological condition characterized by recurrent seizures [1, 2] which account for 1% of the global burden of disease [3]. It affects at least 100 million people worldwide, specifically in childhood and adolescence [4] and 1 in 26 of the population may develop seizures at some point in their lives [5]. The vast majority of them live in low- and middle-income countries [6] and its burden in low-income countries is more than twice that found in high-income countries because of the higher risk factors such as poverty, higher rate of infectious diseases, and brain injuries [7].

A community-based study done in 5 African countries including Kenya, Tanzania, Uganda, Ghana, and South Africa showed the prevalence of epilepsy ranging between 7 and 15 per 1000 people [8]. Likewise, in a study done on older children in rural Kenya, the adjusted prevalence estimates of a lifetime and active epilepsy were 41/1000 and 11/1000, respectively [9]. Moreover, very high prevalence rates of epilepsy have been found in the Zay society Ethiopia with a prevalence of 29.5/1000 [10].

Although seizures are the most common clinical manifestation of epilepsies, individuals with epilepsy are at risk of numerous health problems, include cognitive problems, mental health conditions, including depression, anxiety, and somatic co-morbidities. For many individuals with epilepsy, the co-morbidities are more burdensome than the seizures that cognitive abnormalities are among the most common and troublesome [11].

Cognitive impairment is a significant cognitive decline from a previous level of performance or functioning in one or more domains cogitation (complex attention, executive function, learning, and memory, language, perceptual-motor, or social cognition) [12, 13].

Cognitive impairment is a frequent feature of different types of seizure disorder. Nearly 70% of patients with TLE have problems in declarative memory function, which represents the most common cognitive impairment in this group. Impairment of executive function and low intelligence levels are also quite often observed in about 30% of the patients with TLE [14]. In newly diagnosed and untreated epileptic patients, cognitive problems are already present in more than 50% of patients [13]. Likewise, cognitive impairment has been reported in around 30%–40% of epileptic patients [15].

In another study, between 20–50% of patients with epilepsy have memory impairment [16]. In a study done on newly onset untreated epileptic patients, 49.4% of the participants experienced impairment in attention or executive function, 47.8% impairment in episodic memory, and 39.3% subjective deficits in memory, and 35.2% subjective deficits in attention [17]. Research done in the USA using MMSE among participants living with epilepsy cognitive impairment was found in all domains of cognitive function, including 39.5% with impairment in visual memory, 23.7% each in attention and executive function, 18.4% in visuospatial skills, and 15.8% for both verbal memory and language [18]. Magnitude of cognitive impairment among patients with epilepsy has been evident with studies conducted in Indonesia 69.2% [19], India 36% [20], Burkina Faso 61.8% [21], South-Eastern Nigeria 19.6% [22], Pakistan 39.5% [23] and Ethiopia 26.92% [24]. Cognitive impairment further complicated to difficulty in performing daily day-to-day activities, such individuals may complain of impaired attention, word-finding difficulty, verbal fluency difficulty, forgetfulness, and psychomotor slowing [25]. Thus, epileptic patients with cognitive impairment will have poor quality of life [26].

A variety of factors contribute to cognitive adverse effects (CAE) of epilepsy, including clinical factors (history of medical illness, previous history of mental illness, depression, and anxiety brain insult), seizure-related factors (seizure frequency, duration of seizure disorder, seizure type and age at seizure onset), and antiepileptic medications, and substance-related medications [16, 27–29]. Studies of the cognitive sequelae of epilepsy in Africans including Ethiopia are generally lacking.

Therefore, this study will add a body of knowledge about the magnitude and factors associated with cognitive impairment in patients living with epilepsy. It is also important to provide baseline information for policy-makers and health care managers to integrate mental health services with the primary health care system to screen and manage cognitive impairment among patients with epilepsy.

Thus, this study was intended to assess the cognitive adverse effects and associated factors among epileptic patients attending South Gondar Zone Hospitals Amhara region, Ethiopia 2020/2021.

## Methods and materials

### Study design, period, and setting

A multi-centered institutional-based cross-sectional study was conducted at South Gondar Zone hospitals from December 2020 to January 2021. Debre Tabor is the capital city of the South Gondar zone which is located about 666km, North of Addis Ababa. There are 8 hospitals in this zone which include Debre Tabor comprehensive specialized hospital, Andabet, Mekane-Eyesus, Addis Zemen, Ebnat, Nefas Mewucha, Dr. Ambachew Memorial, and Simada primary hospitals. There were about 1101 patients with epilepsy who visit the outpatient department of South Gondar Zone hospitals per month.

### Sample size determination

The sample size was determined by using a single population proportion formula. Taking into account the following assumption, the proportion (p) is 26.92%; (taken from research conducted on cognitive impairments of epilepsy at Black-Lion neurology clinic, Addis Ababa, Ethiopia [24]), the margin of error (0.05); level of confidence (95%), and non-response rate 10% giving the sample size of 334.

We also calculated the sample size based on factors by taking four variables including duration of seizure disorder, seizure frequency, type of medication, and age at seizures onset giving the sample size of 447, 389,369,542 respectively. Taking the larger sample size, the final size to be 542.

### Study participants and sampling procedures

This research was conducted in eight (8) South Gondar Zone governmental hospitals among patients living with epilepsy including Debre Tabor comprehensive specialized Hospital, Andabet, Estie, Addis Zemen, Ebnat, Lay Gaynt, Dr.Ambachew Memorial, and Simada primary hospitals. All epileptic patients attending outpatients departments were the source populations and those age 18 years and above were included. Those patients with epilepsy who had a co morbid intellectual disability and acutely sick were excluded. A systematic random sampling technique was applied. The sample size was distributed to each hospital proportional to the numbers of epileptic patients attending the outpatient department per month in each of the hospitals. Then interval k was calculated and the first participant was determined by the lottery method.

Thus, K = N/n…1101/542 ~ 2. Finally, the participants were interviewed every 2 intervals

**Fig 1:**
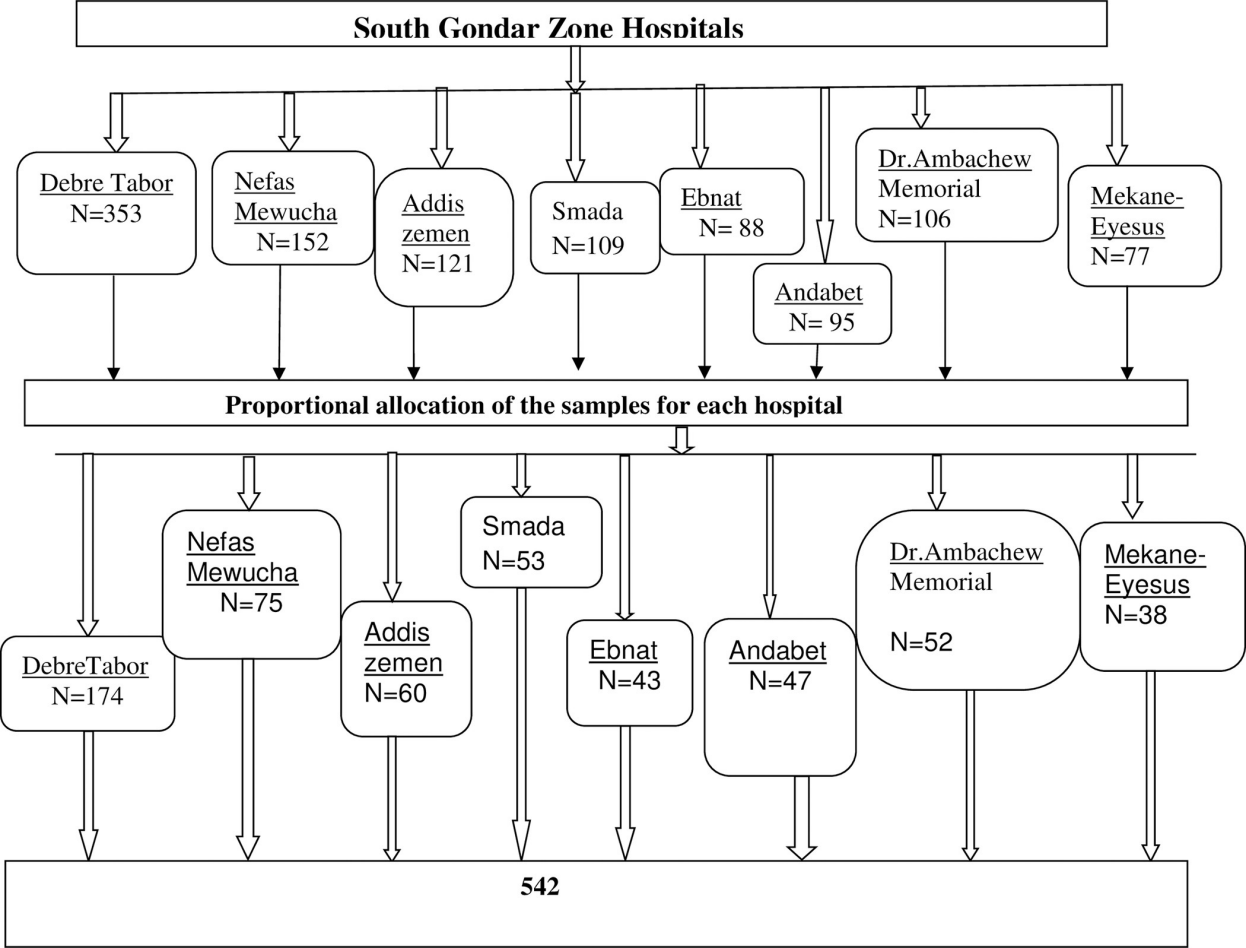
Sampling procedure for the cognitive adverse effect of epilepsy and its predictors attending South Gondar hospitals Amhara Ethiopia 2020/2021.

Of all invited (542) participants, twenty five (25) of the eligible participants refused to participate and eight (8) of the questionnaires were discarded because of incomplete data. Finally, 509 participants completed the questionnaires with a response rate of 93.9% (Fig 1).

### Study variables

The dependent variable was cognitive impairment which was measured using MMSE as a dichotomous variable (Yes/No). Independent variables include socio-demographic factors (age, gender, marital status, educational status resident), clinical variables (history of medical illness, history of mental illness, history of head injury, depression, and anxiety), seizure-related factors (seizure frequency, duration of seizure disorder, seizure type, age at seizure onset), medication and substance-related factors (AEDs, substance intake).

### Data collection tools

Data was collected through face-to-face interviews by using a previously adapted standard questionnaire with the Amharic version of the tool. Cognitive adverse effects of epilepsy were measured using MMSE which had sensitivity and specificity of 87% and 82%, respectively and the MMSE has shown a high degree of correlation with a variety of gold standards tools including MoCA and DSM clinical diagnosis and had enter data reliability 0.89 (Cronbach α) [30].

#### Cognitive impairment

According to the Mini-Mental State Examination tool (MMSE), individuals living with epilepsy who scored less than 25 out of the total score of 30 were considered to have a deficit in cognition [31].

#### Depression and anxiety

Depression and anxiety were screened using HADS in which those individuals who scored > = 8 on HADS were categorized as having depression and anxiety [32].

Current use: using at least one of a specific substance for non-medical purposes within the last three months (alcohol, khat, tobacco, others).

Ever use of a substance: using at least one of any specific substance for a non-medical purpose at least once in the lifetime (alcohol, khat, tobacco, others) [33].

#### Medical illness

To examine a history of medical illness, respondents were asked: ‘Did you have any medical illness (DM, HTN, HIV/AIDS, etc.?)’ and responses were yes/no.

#### Intellectual disability

It has been screened using DSM-V which is defined by significant limitations in both intellectual functioning (reasoning, learning, and problem solving) and in adaptive behaviour (conceptual, social, and practical skills) and it has been screened using DSM-V [34].

### Data quality control and data collectors

Investigators selected 24 Bsc psychiatry professionals working in the eight hospitals as the data collector three for each hospital. The training was given to the data collectors and supervisors on how to properly utilize the data collection tools. The questionnaire was translated into Amharic and then back into English to check its consistency.

A week before the actual data collection, pretest was conducted on 5% (28) of samples from Mekane-Eyesus hospital to check the clarity of the instrument that the data obtained from this was not included in the main analysis part. Based on the finding from the pretest, the questionnaire was revised and adapted especially on the structured questionnaire. Once the participants agreed to participate; they were given pieces of information and signed the informed consent.

Supervision was held regularly during data collection. The collected data were checked daily for completeness and consistency.

### Data processing and analysis

Data were entered into Epi data version 4.4.2 and then export to SPSS version 24 for further statistical analysis. Frequency tables and diagrams were used for presenting the descriptive results.

Bivariable analysis was used to look for the association between predictors and dependent variables and multivariable logistic regression analysis was done to control for confounding and identify the most important associated variables. The strength of associations was indicated by AOR with a 95% confidence interval. P-value < 0.05 was considered statistically significant.

Hosmer and Lemeshow’s test (p = 0.208) was used to check model fitness. Multi-co linearity was checked to see the correlation among the independent variables by using variance inflation factor and tolerance. In this case, the value of variance inflation factor was <10 and tolerance was greater than 0.1 which indicated that there was no dependency between independent variables.

### Ethical consideration

The ethical clearance was obtained from the ethical review committee of Debre Tabor University, and a permission letter was obtained from each hospital. We received written informed consent from study participants and confidentiality was maintained by omitting personal identifiers.

The purpose of the study, direct and indirect advantages of being included in the study were explained to the participants. This form indicated that participation was voluntary and that clients had the right to withdraw from completing the questionnaire at any time they wish.

Participants were also informed that there was no expectation of any benefits for them associated with participating in the study but those who had cognitive impairment on the MMSE scale got appropriate intervention timely.

## Results

### Socio-demographic characteristics

In this study, a total of 509 individuals were included with a response rate of 93.9%. The majority of the respondents were males 312(61.3%) and in the age group between 18–29 years, Most of them were orthodox followers 341(67%), and Amhara by ethnicity 485(95.2%). About 172(33.8%) of them couldn’t read and write, and nearly half of them were the rural residents 251(49.3%) (Table 1).

**Table 1:** Socio-demographic characteristics of patents with epilepsy attending outpatient department of South Gondar zone hospitals Amhara, Ethiopia, 2021(n = 509).

Characteristics	Category	Frequency	Percent
**Sex**	Male	312	61.3
	Female	197	38.7
**Age**	18–29	295	58.0
	30–39	99	19.4
	40–49	49	9.6
	> = 50	66	13
**Ethnicity**	Amhara	485	95.2
	Tigray	13	2.6
	Oromo	11	2.2
**Religion**	Orthodox	341	67
	Muslim	85	16.7
	Protestant	14	2.8
	Catholic	69	13.5
**Residence**	Rural	251	49.3
	Urban	258	50.7
**Educational status**	Unable to read and write	173	34
	primary (1–8)	58	11.4
	High school (9–12)	128	25.1
	College and above	150	29.5

### Clinical factors

In this study, about 89(17.5%) of them had a history of medical illness, 72(14.1%) had a history of head injury, 305 (59.9%) of them had mild to severe depression and 220(43.2%) of them were anxious.

### Seizure and medication-related factors

The majority of participants were diagnosed for general tonic-clonic seizure 191(37.5%) and were treated with either Phenobarbital or Phenytoin 213(41.9%) (Table 2).

**Table 2:** Seizure and medication-related factors of cognitive impairment in patients with epilepsy attending outpatient department of south Gondar zone hospitals Amhara Ethiopia 2021(n = 509).

Characteristics	Category	Frequency	Percent
**Age at seizure onset**	<10	278	54.6
	10–19	91	17.9
	20–29	45	8.8
	30–39	95	18.7
**Duration of the seizure disorder**	<10	315	61.9
	10–29	136	26.7
	> = 30	58	11.4
**Frequency of seizures**	Daily to every other day	146	28.7
	Weekly to every other week	45	8.8
	Once in three to four weeks	116	22.8
	Once in the past 1–6 months	100	19.6
	6–11 months ago	65	12.8
	1–4 years ago	37	7.3
**Type of seizure**	Simple partial	96	18.9
	Focal with secondary		
	generalization	66	13
	Complex partial	109	21.4
	GTCs	191	37.5
	Atonic	28	5.5
	Myoclonic	19	3.7
**Type of AEDs**	Carbamazepine	79	15.5
	Phenobarbital /Phenytoin	213	41.9
	Na+ Valporate	72	14.1
	Phenobarbital +Phenytoin	145	28.5

### Substance-related factors

**Fig 2:**
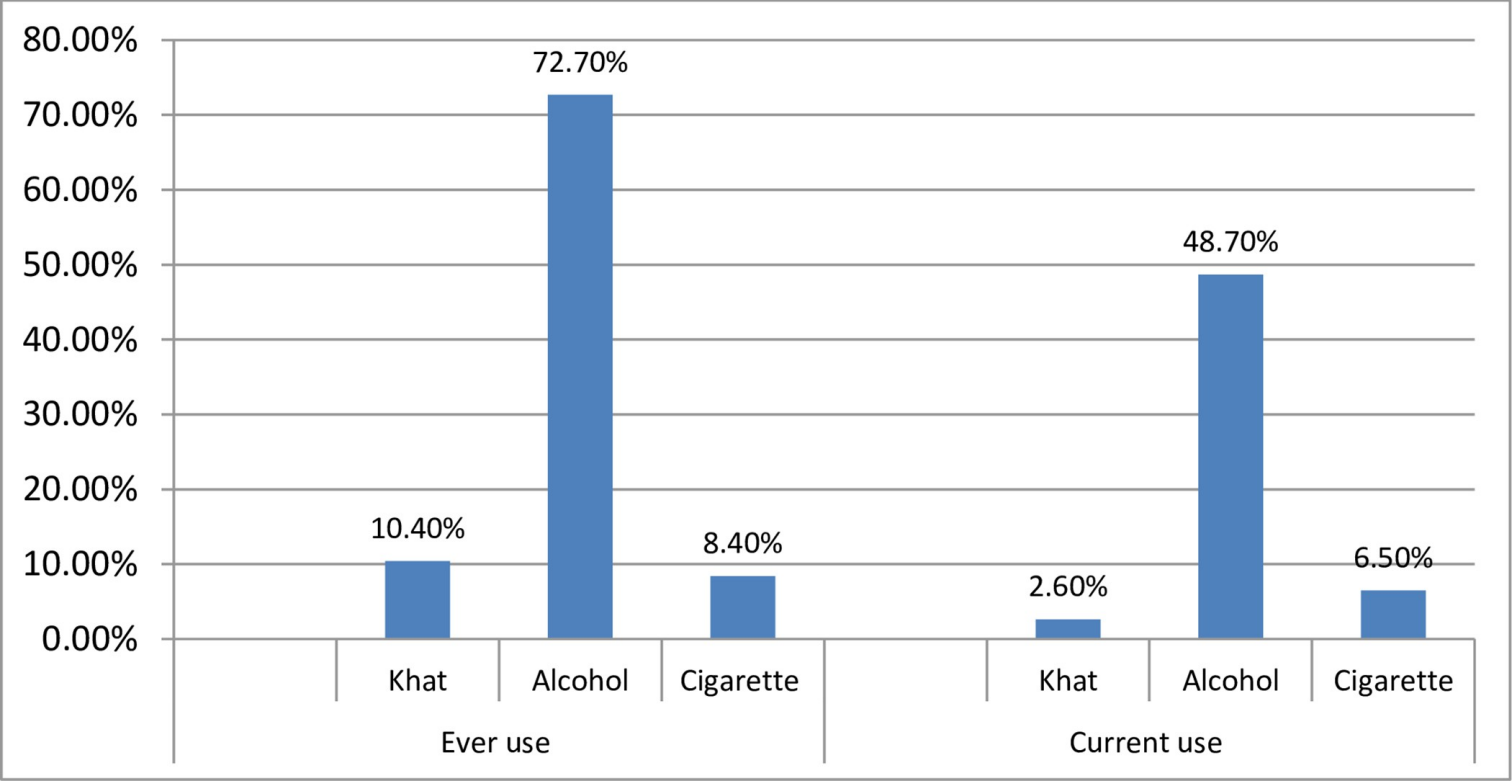
Substance-related factors of cognitive impairment in patients with epilepsy attending outpatient department of south Gondar zone hospitals Amhara Ethiopia 2020 /2021(n = 509).

In this study majority of the respondents had ever use 370(72.7%) and the current use of 248(48.7%) alcohol respectively (Fig 2).

### Prevalence and associated factors of cognitive impairment

In this study magnitude of cognitive impairment among epileptic patients was 69.2% (95CI; 65.4, 73.1).

To determine the association of independent variables with cognitive impairment, bivariable, and multivariable binary logistic regression analyses was carried out.

On the bivariate analysis respondents who are rural residents, unable to read and write, duration of seizure disorder> = 30 years, respondents who took combined Phenobarbital and Phenytoin, respondents who had a history of medical illness, head injury, depression, and anxiety were significantly associated with cognitive impairment at p-value <0.05.

These variables were taken to multivariable analysis to control confounding effects. In multivariable analysis, rural residents, respondents who could not read and write, participants who lived with the seizure disorder for > = 30 years, and who had a history of head injury, participants who took combined Phenobarbital and Phenytoin, having depression and anxiety were significantly associated with cognitive impairment.

When controlling for other variables, the odds of developing cognitive impairment among epileptic patients were 4.59 times higher among those participants who had seizure disorders> = 30years as compared with those who had <10 years duration of seizure disorder (AOR = 4.59, 95%CI; 2.01,10.52). Participants who took combined Phenobarbital and Phenytoin were more affected by cognitive impairment as compared with those participants who took Carbamazepine (AOR = 2.03, 95%CI; 1.21, 4.32). The likelihood of developing cognitive impairment was greater among participants who had a history of head injury (AOR = 3.29, 95%CI; 1.30, 8.32), depression (AOR = 4.76, 95%CI; 2.83, 7.98), and anxiety (AOR = 3.11, 95%CI; 1.58, 6.12) as compared with their counterparts. Moreover, participants who were rural residents (AOR = 4.16, 95%CI; 1.99, 8.67) and could not read and write (AOR = 2.62, 95%CI; 1.24, 5.51) scored lower on the MMSE test (Table 3).

**Table 3:** Factors associated with cognitive impairment among patients with epilepsy attending outpatient department of South Gondar hospitals 2020(n = 509).

Characteristics	Category	Cognitive impairment		COR(95%CI)	AOR(95%CI)
		Yes	No		
Residence	Rural	202	49	*2.97(1.99,4.42)	*4.16(1.99,8.67)
	Urban	150	108	1	1
Educational status	Unable to read & write	154	19	4.05(2.26,7.28)	2.62(1.24,5.51)
	Primary school	45	13	1.73(0.86,3.50)	0.43(0.16,1.18)
	Secondary school	53	75	0.35(0.22,0.58)	0.28(0.14,0.55)
	college and above	100	50	1	1
Duration of seizure disorder	<10	207	108	1	1
	10–29	98	38	1.35(0.87,2.09)	1.51(0.51,4.44)
	> = 30	47	11	2.23(1.11,4.47)	4.59(2.01,10.52)
Type of AEDs	Carbamazepine	61	18	1	1
	Phenobarbital /Phenytoin	139	74	0.55(0.31,1.01)	0.26(0.58,2.73)
	Na+ Valporate	25	47	0.16(0.08,0.32)	1.97(0.70,5.50)
	Phenobarbital +Phenytoin	127	18	2.08(1.01,4.28)	4.69(1.88,11.69)
History of head injury	Yes	63	9	4.06(1.89,8.69)	3.29(1.30,8.32)
	No	289	148	1	1
History of medical illness	Yes	79	10	4.25(2.14,8.46)	0.82(0.29,2.25)
	No	273	147	1	1
Depression	Yes	256	49	5.88(3.89,8.87)	4.76(2.83,7.98)
	No	96	108	1	1
Anxiety	Yes	191	29	5.24(3.32,8.25)	3.11(1.58,6.12)
	No	161	128	1	1

Note that: Hosmer Lemshow test -0.208, Tolerance >0.1, variance inflation factor<10

## Discussion

Most people with epilepsy in low and middle income countries do not seek medical treatment for their epilepsy and all types of epilepsy frequently experience cognitive and emotional difficulties. This study aimed to assess the magnitude of cognitive impairment in epileptic patients and was investigated in relation to the socio-demographic, and other factors which greatly affect the higher executive brain function. This study showed that the magnitude of cognitive impairment was found to be 69.2% (95%CI; 65.4, 73.1). Factors, including living in a rural area, not being able to read or write, having a history of head injury, having a seizure disorder for > = 30 years, using combined phenobarbital and phenytoin, having depression and anxiety were significantly associated with cognitive impairment.

The result of the current study was in line with the study carried out in Indonesia 69.2% [19] but higher than the studies done in the USA 39.5% [18], India 36% [20], Slovakia 37% [35], Burkina Faso 61.8% [21], South-Eastern Nigeria 19.6% [22], Pakistan 39.5% [23] and Ethiopia 26.92% [24]. The difference might be the variation in the tool, socio-cultural differences, the ages of the participants included, and sample size. For example, in Indian and Nigerian studies the sample size was 100 and 102 respectively which were much lower than the current study. Moreover, in the current study MMSE was used but CSID was utilized in the, Nigeria study.

In the contrary, the current study was lower than the Tunisians study (100% of cases had cognitive problems) [36]. The reason could be the difference in the type of seizure disorder included in the studies. In the Tunisian study, the patients were only those with temporal lobe epilepsy but in the current study, all types of seizure disorders were included.

On the independent predictors of cognitive impairment, rural residents, participants who could not read and write, longer duration of illness, taking combined Phenobarbital and Phenytoin, having a history of head injury, those participants who had depression and anxiety scored lower on the MMSE test compared with their counterparts. Participants who had > = 30 years duration of illness were more affected to develop cognitive impairment compared with <10 years duration of illness. This was supported by Merkans (Ethiopia) [24], and USA [37] studies. Likewise, those participants who took combined Phenobarbital and Phenytoin were highly affected by cognitive impairment. This was in agreement with the previous study [38]. The current study also revealed that participants who were positive for depression scored lower on the neuropsychiatric test(MMSE) which was supported by the previous work [39]. Studies showed that depression decrease the volume of bilateral hippocampi, alters the cortical thickness and a reduction of neuronal cell density in the frontal lobe. These results illustrated the negative effects of depression on the cognitive function of epileptic patients [40–42].

Higher anxiety was also negatively associated with cognitive function which was supported by the USA [18], and Slovakia studies [35]. Likewise, respondents with a history of head injury scored lower on MMSE which was supported by the study conducted in Australia [43]. This could be, head injury is the risk for seizure disorder i.e. leads frequent seizure which in turn contributes for the development of cognitive impairment compared with those who had no history of head injury. Participants who were rural residents and could not write and read scored poor cognition in this test. This might be because the MMSE examination is affected by the educational status that overestimates the cognitive impairment in the lower schooling population [44]. Moreover, people who lived in rural areas and those who couldn’t read and write might not get enough information about the treatment of epilepsy so that they become late in seeking help. Therefore, living with untreated seizures for a longer duration exposes poor cognitive performance.

These calls for neuropsychological evaluation and measuring the cognitive status of patients with epilepsy early in the course of the disease. This also indicated the necessity of regular screening of the cognitive side effects of antiepileptic drugs and co morbid disorders in the course of the disease and long term therapy using screening tools.

## Limitations

People with epilepsy may have recall bias that they may face trouble in recalling the onset and the duration of the illness. Additionally, social desirability bias may be a problem since data collection method was face to face interview which force interviewees to give socially acceptable responses especially in case of substance related questions.

Moreover, the MMSE is affected by educational status i.e. participants with lower educational status might show lower on the test and vice versa.

## Conclusion

The prevalence of cognitive impairment was found to be high. Longer duration of seizure disorder, taking combined Phenobarbital and Phenytoin, having a history of head injury, respondents who were rural residents, who couldn’t write and read, those participants who had depression and anxiety were greatly affected by cognitive impairment compared with their counterparts. Therefore, emphasis should be given to those rural residents, with history of head injury, couldn’t read and write, on increasing the availability of second-generation AEDs and avoidance of routine prescriptions of combined old generation medications like phenytoin and phenobarbital at a time. Similarly, communities’ health education regarding the treatment of epilepsy is crucial, especially for those rural residents and participants who couldn’t read and write. Moreover, integration for routine screening of patients for depression and anxiety is recommended for early prevention of cognitive impairment.
